# Lethal and Sublethal Effects of Insecticides Used on Citrus, on the Ectoparasitoid *Tamarixia radiata*

**DOI:** 10.1371/journal.pone.0132128

**Published:** 2015-07-01

**Authors:** Vitor Hugo Beloti, Gustavo Rodrigues Alves, Diogo Feliciano Dias Araújo, Mateus Manara Picoli, Rafael de Andrade Moral, Clarice Garcia Borges Demétrio, Pedro Takao Yamamoto

**Affiliations:** 1 Department of Entomology and Acarology, “Luiz de Queiroz” College of Agriculture/University of São Paulo (ESALQ/USP), Piracicaba, São Paulo, Brazil; 2 Department of Biology, Faculty of Philosophy, Sciences and Letters of Ribeirão Preto/University of São Paulo (FFCLRP/USP), Ribeirão Preto, São Paulo, Brazil; 3 Department of Agricultural Statistics and Experimentation, “Luiz de Queiroz” College of Agriculture/University of São Paulo (ESALQ/USP), Piracicaba, São Paulo, Brazil; CNRS, FRANCE

## Abstract

Huanglongbing (HLB) is a disease associated with the bacteria “*Candidatus* Liberibacter spp.” and has been devastating citrus orchards around the world. Its management involves control of the insect vector, the Asian citrus psyllid *Diaphorina citri* Kuwayama. However, the indiscriminate use of chemicals has caused pest outbreaks and eliminated the natural enemies of the vector, such as the parasitoid *Tamarixia radiata* (Waterston), the main agent for biological control of *D*. *citri*. This study assessed the lethal and sublethal effects of insecticides recommended for integrated production of citrus on the parasitoid *T*. *radiata*. When adult parasitoids were exposed to residues of 25 insecticides, 20% of them, i.e., gamma-cyhalothrin, etofenprox, azadirachtin, tebufenozide and pyriproxyfen, were considered as harmless (Class 1), 12% as slightly harmful (Class 2), 12% as moderately harmful (Class 3) and 56% as harmful (Class 4), according to the classification proposed by the IOBC/WPRS. Afterward, 14 insecticides (5 harmless and 9 harmful) were sprayed on the parasitoid pupae. Of the 14 insecticides tested, only the organophosphates dimethoate and chlorpyrifos affected the parasitoid emergence. The effects of insecticides on the parasitism capacity of adults exposed to residues of azadirachtin, etofenprox, gamma-cyhalothrin, pyriproxyfen and tebufenozide (harmless) were also evaluated. Tebufenozide and gamma-cyhalothrin affected the parasitism of the F_0_ generation, but did not affect the emergence of the F_1_ and F_2_ generations. Therefore, for an effective IPM program, selective insecticides or harmful pesticides to adult parasitoids could be used in the field, provided that the adults do not occur naturally and the chemical applications do not coincide with parasitoid releases.

## Introduction

The incidence of Huanglongbing (HLB), or greening, a disease associated with the bacteria “*Candidatus* Liberibacter asiaticus” and “*Candidatus* Liberibacter americanus” and transmitted by the Asian citrus psyllid *Diaphorina citri* Kuwayama (Hemiptera: Liviidae), has increased since 2004, when HLB began to be reported in the main states producing citrus for juice, in São Paulo, Brazil [1,2] and in Florida, USA [3]. Researchers consider this disease as the major threat to world citriculture [4].

Since there are no curative measures for the disease and resistant varieties have not yet been discovered, the management of HLB is based on the use of healthy nursery trees, elimination of HLB-symptomatic plants, and especially on the control of the insect vector *D*. *citri* [5]. Among the tactics for management of the insect vector, citrus growers have mostly used insecticide sprays [6]. Although effective, this tactic can change the biological balance of an agro-ecosystem due to the occurrence of secondary pest outbreaks, resurgence of target pests, selection of resistant populations, and biological imbalances [7,8], which increase production costs and reduce the effectiveness of the technique and the environmental sustainability of the system [9].

Thus, conservation and increase of biological control agents have become an important strategy to reduce population levels of pests and impacts caused by overuse of agrochemicals. Biological control, used for the management of *D*. *citri* in several countries, is based on the release of parasitoids in non-commercial citrus plantings and in areas where alternative hosts of the insect vector occur [10]. Among the natural enemies, the ectoparasitoid *Tamarixia radiata* (Waterston) (Hymenoptera: Eulophidae) and the endoparasitoid *Diaphorencyrtus aligarhensis* (Shafee, Alam and Agarwald) (Hymenoptera: Encyrtidae) [11] are the most important. *T*. *radiata* has been more efficient in the control of *D*. *citri* and is used in biological control programs in several countries, including Brazil [10].

*T*. *radiata* is native to India, and is an idiobiont ectoparasitoid specific for *D*. *citri* [12]. It develops preferably on the 3^rd^ to 5^th^-instar nymphs of the psyllid [13]. *T*. *radiata* can also feed on eggs and nymphs from the 1^st^ to 3^rd^ instars [14]. Due to the combined effect of feeding and parasitism, a single female of *T*. *radiata* is believed to eliminate up to 500 nymphs of the psyllid throughout its life [14]. On Reunion Island, *T*. *radiata* achieved a 70% incidence of parasitism of nymphs of *D*. *citri* [15].

The successful use of this parasitoid depends not only on adequate decision-making on the part of the professionals involved in the management of HLB, but also on the tools for its implementation, such as the availability of selective insecticides for pest management. However, little information is available in the literature about the lethal and sublethal effects of insecticides on *T*. *radiata*, and some studies, such as that of Hall and Nguyen [16], are restricted to the few agrochemicals used in citrus. Few studies are available even with other species of *Tamarixia*; Martinez et al. [17] researched the effect of three pesticides on *Tamarixia triozae* (Burks) (Hymenoptera: Eulophidae).

In view of the importance of using the parasitoid *T*. *radiata* in IPM programs for *D*. *citri*, this study evaluated the acute toxicity and sublethal effects of insecticides recommended for the control of insect pests on adults and pupae of *T*. *radiata*, in order to contribute to IPM programs that use both chemical control and parasitoid releases.

## Material and Methods

### Insects

The insects used in this study were obtained from the maintenance colony of the Insect Biology Laboratory and the Integrated Pest Management Laboratory in the Department of Entomology and Acarology of the Luiz de Queiroz College of Agriculture, University of São Paulo (ESALQ/USP).

The parasitoids *T*. *radiata* were reared in a room with the temperature controlled at 25 ± 2°C, relative humidity (RH) 70 ± 10% and a photoperiod of 14 L: 10 D h. For rearing, seedlings of orange jasmine [*Murraya paniculata* (L.) Jack (Rutaceae)] grown in 2-L pots were used. The seedlings were first pruned to a height of 25 cm and, after producing sprouts (2–3 cm long), they were placed in rearing cages (40 × 60 × 50 cm) and used as a substrate for female oviposition and food for *D*. *citri* nymphs, according to the method proposed by Nava et al. [18]. When the *D*. *citri* nymphs reached the 4^th^ and/or 5^th^ instars, the seedlings of *M*. *paniculata* were transferred to acrylic cages (90 × 50 × 50 cm) and adults of *T*. *radiata* were released to parasitize the nymphs, in accordance with the method described by Gómez-Torres et al. [19]. Honey drops were placed on the sides of the cages to feed the adult parasitoids.

### Insecticides

Twenty-five insecticides recommended for integrated citrus production (PICitrus) in Brazil and registered for insect pest control were assessed on the parasitoid *T*. *radiata*. All products were tested at the highest concentrations recommended by the Brazilian Ministry of Agriculture, Livestock and Supply (MAPA) [20]. The insecticides and dosages used (g i. a. L^-1^) in the bioassays are listed in Table 1.

**Table 1:** Insecticides used in bioassays, with their concentrations and chemical group.

Insecticide	Trade name	Dosage used (g a. i. L^-1^)	Chemical group
Thiamethoxam	Actara 25 WG	0.025	Neonicotinoid
Cypermethrin	Akito	0.025	Pyrethroid
Chlorantraniliprole + Lambda-cyhalothrin	Ampligo	0.02 + 0.01	Anthranilamide + Pyrethroid
Buprofezin	Applaud	0.5	Thiadiazinone
Azadirachtin	Azamax	0.03	Tetranortriterpenoide
Deltamethrin	Decis Ultra 10 EC	0.0075	Pyrethroid
Formetanate	Dicarzol	0.15	Phenyl Methylcarbamate
Lambda-cyhalothrin + thiamethoxam	Engeo Pleno	0.02 + 0.02	Pyrethroid + Neonicotinoid
Imidacloprid	Evidence 70 WG	0.03	Neonicotinoid
Phosmet	Imidan 50 WP	0.25	Organophosphate
Lambda-cyhalothrin	Karate Zeon 5 CS	0.01	Pyrethroid
Chlorpyrifos	Lorsban 48 BR	0.72	Organophosphate
Malathion	Malathion 100 EC	1.5	Organophosphate
Tebufenozide	Mimic 24 SC	0.12	Diacylhydrazine
Gamma-cyhalothrin	Nexide	0.0075	Pyrethroid
Mineral Oil	Mineral Oil Argenfrut	8.46	Aliphatic Hydrocarbon
Vegetable Oil	Vegetable Oil Nortox	9.30	Fatty Acid Esters
Dimethoate	Perfekthion	0.8	Organophosphate
Imidacloprid	Provado 20 SC	0.04	Neonicotinoid
Acetamiprid	Saurus	0.06	Neonicotinoid
Esfenvalerate	Sumidan 15 SC	0.02	Pyrethroid
Pyriproxyfen	Tiger	0.006	Ether Pyridine
Spinosad	Tracer	0.07	Spinosyns
Etofenprox	Trebon 10 SC	0.025	Pyrethroid Ether
Beta-cyfluthrin	Turbo	0.012	Pyrethroid

### Bioassays

All bioassays were conducted in a room with the temperature regulated at 25 ± 2°C, RH 70 ± 10% and a photoperiod of 14 L: 10 D h, using a completely randomized design.

### Acute toxicity of insecticides to adults of *Tamarixia radiata*

To evaluate the acute toxicity of insecticides to adults of *T*. *radiata* (the most susceptible stage to insecticides), leaf discs 4.0 cm in diameter of Valencia Sweet Orange [*Citrus sinensis* (L.) Osbeck (Rutaceae)] were sprayed with 2 mL of the insecticide solution for the respective treatment, using a Potter tower (Burkard Scientific, Uxbridge, UK) with the pressure adjusted to 0.7 kg cm^-2^, corresponding to a deposit of 1.8 ± 0.1 mg cm^-2^ of leaf area, in compliance with recommendations of the International Organization for Biological and Integrated Control of Noxious Animals and Plants/West Regional Palearctic Section (IOBC/WPRS). Distilled water was used as the control treatment.

After spraying, the discs were kept in a climate-controlled room for three hours to allow the residue to dry. Afterward, the discs were placed in Petri dishes (4.5 cm diameter) containing a 2.5% agar: water solution to prevent the leaf discs from drying; each dish was considered an experimental unit. Later, 10 adults of *T*. *radiata* up to 48 h old were anesthetized with CO_2_ for 5 s and released in each dish. The dishes were closed with voile fabric, which was affixed to the cover lid, with a hole in the central region to allow gas exchange. On top of the voile fabric, a droplet of honey (~ 1 mm^3^) was placed to serve as food for the parasitoids during the evaluation period of the bioassay. Five repetitions were used for each treatment (*n* = 50).

The mortality was assessed 24 h after exposure of the parasitoids to their treatments, counting live and dead insects in each experimental unit. Insects that responded to the touch of a soft-bristled brush were considered alive. Acute toxicity (mortality) of each treatment was calculated using the formula of Abbott [21]. Based on mortality data, the insecticides were classified according to the rates proposed by the IOBC/WPRS: Class 1 = Mc < 25% (harmless); Class 2 = 25 ≤ Mc ≤ 50% (slightly harmful); Class 3 = 51 ≤ Mc ≤ 75% (moderately harmful) and Class 4 = Mc > 75% (harmful) [22].

### Acute toxicity of insecticides to pupae of *Tamarixia radiata*

To evaluate the acute toxicity of insecticides applied on pupae (most tolerant stage) of *T*. *radiata*, 14 insecticides were selected for the test. Five that were considered harmless (Class 1) to adults of *T*. *radiata* in the acute toxicity test (item 2.3.1) (azadirachtin, etofenprox, gamma-cyhalothrin, pyriproxyfen and tebufenozide) and nine harmful (Class 4) (beta-cyfluthrin, chlorpyrifos, cypermethrin, dimethoate, phosmet, spinosad, esfenvalerate, imidacloprid and thiamethoxam) were chosen. For each treatment, seven repetitions were used; each experimental unit consisted of a Petri dish containing a branch of *M*. *paniculata* with pupae of *T*. *radiata*.

To obtain parasitoid pupae, seedlings of *M*. *paniculata* containing nymphs of the 3^rd^ to 5^th^ instars were selected from the maintenance colony and placed in cages (90 × 50 × 50 cm). Subsequently, 50 adults of *T*. *radiata* were released for parasitism of the nymphs for 48 h, and were then removed and discarded. Nine days after the host-parasitoid contact (egg-adult period according to Rosa et al. [23]) with mummified nymphs, the branches were cut from the orange jasmine plants and sprayed using a Potter tower, as described in section 2.3.1. Distilled water was used as the control treatment.

After the spraying, the branches were transferred to Petri dishes (4.5 cm diameter) containing moistened cotton. Insect emergence was assessed daily. For the emerged adults, life-cycle duration and sex were assessed, with subsequent calculation of the sex ratio. Longevity was also evaluated. For this, in treatments with insect emergence, 30 parasitoids (15 females and 15 males) were placed in individual glass tubes (2.5 cm diameter × 8.5 cm long) closed with PVC plastic film. A honey droplet (~ 1 mm^3^) was placed inside the tubes for insect feeding.

### Effects of insecticides on parasitism rate of *Tamarixia radiata*

Insecticides considered harmless (Class 1) to adults of *T*. *radiata* in the acute toxicity test (item 2.3.1) were tested (Table 2).

**Table 2:** Mean number of *Tamarixia radiata* live adults, Corrected mortality (Cm) and class of toxicity of the compounds according to IOBC/WPRS, under 25 ± 2° C, 70 ± 10% RH and photoperiod 14 L: 10 D h.

Treatments	Chemical group	Live adults M ± SE (n = 50)	Cm (%)	IOBC/WPRS^1^
Control	-	8.2 ± 0.49	-	-
Chlorantraniliprole + Lambda-cyhalothrin	Anthranilamide + Pyrethroid	4.2 ± 0.66	48.8	2
Cypermethrin	Pyrethroid	1.0 ± 0.55	87.8	4
Deltamethrin		4.4 ± 0.93	46.3	2
Lambda-cyhalothrin		4.2 ± 1.32	48.8	2
Gamma-cyhalothrin		6.2 ± 0.86	24.4	1
Esfenvalerate		0.2 ± 0.20	97.6	4
Etofenprox		6.8 ± 1.46	17.1	1
Beta-cyfluthrin		0.4 ± 0.40	95.1	4
Lambda-cyhalothrin + thiamethoxam	Pyrethroid + Neonicotinoid	1.0 ± 1.00	87.8	4
Thiamethoxam	Neonicotinoid	0.0 ± 0.00	100.0	4
Imidacloprid 700 WG		3.2 ± 0.97	60.9	3
Imidacloprid 200 SC		1.8 ± 0.58	78.1	4
Acetamiprid		2.8 ± 0.74	65.9	3
Phosmet	Organophosphate	0.2 ± 0.20	97.6	4
Chlorpyrifos		0.0 ± 0.00	100.0	4
Malathion		0.0 ± 0.00	100.0	4
Dimethoate		0.0 ± 0.00	100.0	4
Formetanate	Phenyl Methylcarbamate	0.6 ± 0.40	92.7	4
Mineral Oil	Aliphatic Hydrocarbon	2.8 ± 1.24	65.9	3
Vegetable Oil	Fatty Acid Esters	2.0 ± 2.00	97.6	4
Spinosad	Spinosine	0.0 ± 0.00	100.0	4
Azadirachtin	Tetranortriterpenoide	6.2 ± 1.20	24.4	1
Buprofezin	Thiadiazinone	0.6 ± 0.40	92.7	4
Tebufenozide	Diacylhydrazine	8.4 ± 1.17	0.0	1
Pyriproxyfen	Pyridine	7.4 ± 1.17	9.8	1

^1^IOBC/WPRS class based on parasitoid mortality: Class 1 = Mc < 25% (harmless); Class 2 = 25 ≤ Mc ≤ 50% (slightly harmful); Class 3 = 51 ≤ Mc ≤ 75% (moderately harmful) and Class 4 = Mc > 75% (harmful) (Van de Veire et al., 2002).

Females of *T*. *radiata* that survived the exposure to insecticide residues were transferred to cages made with voile fabric containing branches of *M*. *paniculata* with 4^th^-instar nymphs, for a period of 48 h for parasitism. Afterward, the females were removed from the cages. Honey droplets (~ 1 mm^3^) were placed on the leaves of *M*. *paniculata* for parasitoid feeding. For each treatment, 11 repetitions were performed, each repetition consisting of a female parasitoid.

From the 9^th^ day after the host-parasitoid contact (egg-adult development period, according to Rosa et al. [23]), the emergence rate was evaluated based on the number of adults emerged from parasitized nymphs. The duration of the egg-adult period of *T*. *radiata* was also determined.

After emergence, couples were formed from the offspring (F_1_ generation) of all treatments and offered *D*. *citri* nymphs again to assess parasitism capacity, as well as to determine the existence of reduced parasitism and possible effects on the F_2_ generation.

For this, a couple from the F_1_ generation was placed in contact for 48 h with 4^th^-instar nymphs that were previously transferred to Rangpur lime seedlings (*Citrus limonia*, Osbeck). Each seedling was transferred to a voile fabric cage. The number of repetitions for each treatment varied (range 5–12), as it depended on the number of insects in the F_1_ generation emergence. Each repetition consisted of a seedling.

As conducted for the F_1_ generation, from the 9^th^ day after host-parasitoid contact, the emergence rate was assessed based on the number of adults emerged from parasitized nymphs. In addition, we evaluated the duration of the life cycle and the longevity of the offspring (F_2_). To evaluate longevity, a sample of 20 insects (10 females and 10 males) of the offspring was evaluated per treatment. For this, the parasitoids were placed in individual glass tubes closed with PVC plastic film, with a honey droplet (~ 1 mm^3^) inside for food. Evaluations were carried out daily to measure the duration of the egg-adult period duration.

The reduction in parasitism capacity was calculated using the formula:

$$R=\left[1-\left(\frac{P}{p}\right)\right]\times 100,$$

where *R* = percentage of reduction in parasitism capacity, *P* = mean value of parasitism for each insecticide, and *p* = mean parasitism observed for the control treatment [24]. Based on the data obtained, the insecticides were classified into categories depending on the toxicological reduction in parasitism capacity, according to IOBC/WPRS recommendations: Class 1 = harmless (R < 30%), Class 2 = slightly harmful (30 ≤ R < 79%), Class 3 = moderately harmful (80 ≤ R ≤ 99%) and Class 4 = harmful (R > 99%) of parasitism capacity reduction [24].

### Data analyses

Generalized linear models [25] of the quasi-binomial type were used for data on the emergence rate and sex ratio (bioassay 2.3.2) and data on the parasitism rate and emergence of the F_1_ and F_2_ generations (bioassay 2.3.3). The quality of the fit was assessed through half-normal probability charts with a simulation envelope [26]. The means were compared through confidence intervals (95%) for linear predictors of the fitted model. An analysis of variance model was adjusted to the duration data, and an analysis of variance model was adjusted to the longevity data, processed with the square root, to meet the model assumptions; the means of both analyses were compared by the Tukey test (p ≤ 0.05). All analyses were conducted in the statistical software “R” version 3.1.2 [27].

## Results

### Acute toxicity of insecticides to adults of *Tamarixia radiata*

The acute toxicity test showed significant differences among the treatments (F_20, 84_ = 8.78, p < 0.0001) (Table 2). Residues of cypermethrin, esfenvalerate, beta-cyfluthrin, lambda-cyhalothrin + thiamethoxam, thiamethoxam, imidacloprid 20 SC, phosmet, chlorpyrifos, malathion, dimethoate, formetanate, vegetable oil, spinosad and buprofezin caused mortality higher than 75%, considered harmful (Class 4) to the adult parasitoids (Table 2). Imidacloprid 70 WG, acetamiprid and mineral oil caused mortalities of 60.9, 65.9 and 65.9%, respectively, considered moderately harmful (Class 3) to adults of *T*. *radiata* (Table 2). Chlorantraniliprole + lambda-cyhalothrin, deltamethrin and lambda-cyhalothrin were considered slightly harmful (Class 2) to adults of *T*. *radiata* (mortality between 46.3 and 48.8%) (Table 2). Gamma-cyhalothrin, etofenprox, azadirachtin, tebufenozide and pyriproxyfen caused less than 25% mortality and were considered harmless (Class 1) to adults of *T*. *radiata* (Table 2).

### Acute toxicity of insecticides to pupae of *Tamarixia radiata*

When pupae of *T*. *radiata* were sprayed with insecticides, significant differences were observed in the emergence rate (F_8, 54_ = 9.48; p < 0.0001) and in the duration of the egg-adult cycle (F_14, 83_ = 3.81; p < 0.0001). The sex ratio (F_13, 73_ = 1.53; p = 0.1258) and longevity (F_10, 213_ = 1.66; p = 0.0917) were not affected by the insecticides (Table 3). The small number of insects that emerged when the parasitoid pupae were treated with chlorpyrifos did not allow us to calculate the sex ratio and longevity for this treatment.

**Table 3:** Emergence percentage, cycle duration, sex ratio and longevity of the parasitoid *Tamarixia radiata* sprayed on the 9th day of development (pupal stage), under 25 ± 2°C, 70 ± 10% RH and photoperiod 14 L: 10 D h.

Treatment	Emergence (%)^1^	Duration egg-adult (days)^2^	Sex Ratio^1^	Longevity (days)^2^
Thiamethoxam	100.0 ± 0.00*	9.8 ± 2.31 bcd	0.3 ± 0.09 a	5.0 ± 0.00*
Cypermethrin	95.2 ± 4.76 a	14.9 ± 0.77 ab	0.3 ± 0.09 a	6.1 ± 0.59 a
Azadiracthin	90.3 ± 3.56 a	10.7 ± 1.20 abcd	0.3 ± 0.07 a	4.3 ± 0.48 a
Phosmet	100.0 ± 0.00*	10.5 ± 1.96 abcd	0.3 ± 0.08 a	6.7 ± 1.46 a
Chlorpyrifos	18.6 ± 11.22 b	4.8 ± 1.63 d	0.0 ± 0.00*	-*
Tebufenozide	88.1 ± 6.69 a	10.9 ± 0.84 abcd	0.4 ± 0.07 a	5.7 ± 0.61 a
Gamma-cyhalothrin	97.8 ± 1.42 a	7.3 ± 1.67 cd	0.4 ± 0.14 a	4.5 ± 0.54 a
Dimethoate	40.9 ± 17.89 b	13.2 ± 1.89 abc	0.1 ± 0.07 a	4.0 ± 0.41 a
Imidacloprid 20 SC	100.0 ± 0.00*	13.8 ± 0.51 abc	0.3 ± 0.05 a	1.0 ± 0.00*
Esfenvalerate	100.0 ± 0.00*	14.0 ± 0.95 abc	0.2 ± 0.06 a	5.1 ± 0.82 a
Pyriproxyfen	91.7 ± 3.72 a	10.6 ± 1.18 abcd	0.5 ± 0.12 a	4.6 ± 0.54 a
Spinosad	87.8 ± 12.24 ab	17.9 ± 2.54 a	0.3 ± 0.16 a	0.5 ± 0.00*
Etofenprox	94.6 ± 3.80 a	11.3 ± 1.18 abcd	0.3 ± 0.09 a	4.1 ± 0.35 a
Beta-cyfluthrin	100.0 ± 0.00*	8.8 ± 1.85 bcd	0.4 ± 0.10 a	5.8 ± 1.40 a
Control	100.0 ± 0.00*	10.0 ± 0.96 bcd	0.3 ± 0.05 a	4.0 ± 0.34 a

*Treatments excluded from the analysis since there was no variability.

^1^Means followed by the same letter in a column do not differ significantly; *p* ≤ 0.05.

^2^Means followed by the same letter in a column do not differ significantly, by Tukey test; *p* ≤ 0.05.

Only the dimethoate and chlorpyrifos treatments negatively affected parasitoid emergence, and the spinosad treatment prolonged the duration of the life cycle (egg-adult) of the parasitoid, in addition to reducing the longevity of the offspring (Table 3).

### Sublethal effects of insecticides on adults of *Tamarixia radiata*

Significant differences were observed in the parasitism rate of the F_0_ generation (F_5, 60_ = 5.00; p < 0.001); the lowest parasitism rates were observed in the tebufenozide and gamma-cyhalothrin treatments (Table 4). The tebufenozide treatment caused the greatest reduction in parasitism capacity of the F_0_ generation, reducing parasitism by 79% in relation to the control treatment. The smallest reduction was observed in the etofenprox treatment, which reduced parasitism by only 35%. However, all insecticides were categorized as slightly harmful (Class 2), according to the toxicological classification categories proposed by the IOBC/WPRS (Table 4).

**Table 4:** Mean parasitism (%), reduced parasitism of *Tamarixia radiata* adults exposed to residues of different insecticides, and toxicological class proposed by the IOBC/WPRS, under 25 ± 2°C, 70 ± 10% RH and photoperiod 14 L: 10 D h.

Treatment	F_0_		
	Mean parasitism ± SE^1^ (%)	Parasitism reduction^2^ (%)	IOBC/WPRS Class^3^
Azadirachtin	19.2 ± 8.48 ab	62	2
Tebufenozide	10.4 ± 5.55 b	79	2
Gamma-cyhalothrin	14.8 ± 7.26 b	71	2
Pyriproxyfen	26.6 ± 11.84 ab	48	2
Etofenprox	33.5 ± 11.10 ab	35	2
Control	50.9 ± 10.59 a	-	-

^1^Means followed by the same letter in a column do not differ significantly, by Tukey test; *p* ≤ 0.05.

^2^Parasitism reduction calculated by the formula *R* = (1-(*P*/*p*))*100.

^3^Toxicological class: class 1 = Harmless (<30% reduction), class 2 = Slightly harmful (30% to 79% reduction), class 3 = Moderately harmful (80% to 99% reduction) and class 4 = Harmful (>99% reduction).

Differences were also observed in the life-cycle duration (egg-adult) of the parasitoids in the F_1_ generation (F_5, 29_ = 16.79; p < 0.0001), and all treatments differed from the control (Table 5). When the reduction in the parasitism capacity was assessed for the F_1_ generation, a reduction was observed in all treatments, although it was smaller than that observed in the F_0_ generation; and only pyriproxyfen and tebufenozide continued to be classified as slightly harmful (Class 2). The other treatments were classified as harmless (Class 1) (Table 5).

**Table 5:** Biological parameters, parasitism and parasitism reduction of *Tamarixia radiata* adults of the F_1_ generation exposed to residues of different insecticides, and toxicological class proposed by the IOBC/WPRS, under 25 ± 2°C, 70 ± 10% RH and photoperiod 14 L: 10 D h.

Treatment	F_1_				
	Emergence rate ± SE^1^ (%)	Duration egg-adult (days)^2^	Mean parasitism ± SE^1^ (%)	Parasitism Reduction^3^ (%)	IOBC /WPRS^4^
Azadirachtin	100.0 ± 0.00*	13.2 ± 0.39 a	64.0 ± 22.27 a	23	1
Tebufenozide	95.8 ± 2.30 a	14.2 ± 0.93 a	50.9 ± 6.82*	48	2
Gamma-cyhalothrin	86.7 ± 13.33 a	12.9 ± 0.36 a	75.3 ± 19.03 a	9	1
Pyriproxyfen	100.0 ± 0.00*	13.3 ± 0.66 a	55.8 ± 11.71 a	33	2
Etofenprox	95.5 ± 3.23 a	13.1 ± 0.30 a	67.8 ± 10.16 a	18	1
Control	93.4 ± 1.40 a	9.9 ± 0.15 b	83.0 ± 9.10 a	-	-

*Treatments excluded from the analysis since there was no variability.

^1^Means followed by the same letter in a column do not differ significantly; *p* ≤ 0.05.

^2^Means followed by the same letter in a column do not differ significantly, by Tukey test; *p* ≤ 0.05.

^3^Parasitism reduction calculated by the formula *R* = (1-(*P*/*p*))*100.

^4^Toxicological class: class 1 = Harmless (<30% reduction), class 2 = Slightly harmful (30% to 79% reduction), class 3 = Moderately harmful (80% to 99% reduction) and class 4 = Harmful (>99% reduction).

There was no statistical difference in the longevity of the offspring of the F_2_ generation (F_5, 85_ = 7.10; p < 0.0001); azadirachtin and tebufenozide increased longevity, differing from the control, with means of 10.2 and 9.6 d, respectively (Table 6).

**Table 6:** Biological parameters of *Tamarixia radiata* adults of the F_2_ generation exposed to residues of different insecticides, and toxicological class proposed by the IOBC/WPRS, under 25 ± 2°C, 70 ± 10% and photoperiod 14 L: 10 D h.

Treatment	F_2_		
	Emergence rate ± SE^1^ (%)	Duration egg-adult (days)^2^	Longevity (days) ^2^
Azadirachtin	60.7 ± 6.07 a	9.7 ± 1.28 a	10.2 ± 1.02 a
Tebufenozide	86.2 ± 6.15*	10.0 ± 0.00*	9.6 ± 0.44 a
Gamma-cyhalothrin	95.8 ± 2.95 a	10.5 ± 0.05 a	5.8 ± 0.65 b
Pyriproxyfen	91.8 ± 4.65 a	10.8 ± 0.23 a	6.8 ± 0.54 b
Etofenprox	86.5 ± 3.29 a	10.8 ± 0.26 a	5.6 ± 0.62 b
Control	85.9 ± 7.12 a	10.2 ± 0.18 a	6.6 ± 0.56 b

*Treatments excluded from the analysis since there was no variability.

^1^Means followed by the same letter in a column do not differ significantly; *p* ≤ 0.05.

^2^Means followed by the same letter in a column do not differ significantly, by Tukey test; *p* ≤ 0.05.

## Discussion

Lethal and sublethal effects of 25 insecticides recommended for integrated citrus production, on the ectoparasitoid *T*. *radiata* were evaluated. The insecticides, belonging to different chemical groups, were classified in different categories proposed by the IOBC/WPRS. The pyrethroids cypermethrin, esfenvalerate and beta-cyhluthrin, the neonicotinoids thiamethoxam and imidacloprid 20 SC, organophosphates, vegetable oil, and spinosad and buprofezin, which belong to the chemical group of thiadiazinone, caused the highest levels of acute toxicity (over 78% mortality) to adults of the parasitoid. These results are similar to those obtained by Hall and Nguyen [16], who observed mortality rates greater than 90% when adults of *T*. *radiata* were exposed to residues of chlorpyrifos and imidacloprid. However, these authors found that vegetable oil caused low mortality of *T*. *radiata*, diverging from the results found in this study. In addition to the effects on *T*. *radiata*, chlorpyrifos, dimethoate, spinosad, esfenvalerate, formetanate and phosmet are also harmful to the parasitoid *Colpoclypeus florus* (Walker) (Hymenoptera: Eulophidae) [28], and malathion caused 100% mortality of the parasitoid *Encarsia* sp. (Hymenoptera: Aphelinidae) [29]. These results indicate that these insecticides can cause high mortality of the parasitoid *T*. *radiata*, hindering its action in biological control of *D*. *citri;* and should be avoided during or even after parasitoid releases in management programs for *D*. *citri*.

The results of exposure of *T*. *radiata* adults to acetamiprid residues were similar to those found by Shankarganesh et al. [30] for adults of the egg parasitoid *Trichogramma chilonis* (Ashmead) (Hymenoptera: Trichogrammatidae) under laboratory conditions, and by Moura et al. [31] for adults of *T*. *pretiosum*. Therefore, these residues were considered moderately harmful in all these studies.

For mineral oil, Hall and Nguyen [16] observed higher mortality than that found in this study when *T*. *radiata* was exposed to product residues. These differences may be associated with the concentration of mineral oil used. Hall and Nguyen [16] used a concentration of mineral oil three times higher than that used in this study. According to Rodrigues and Childers [32], petroleum oils are not selective for natural enemies, but they have a short residual activity. Narrow range mineral oil caused very high mortality on the adults, while a lower acute toxicity was recorded on young instars of *Aphytis melinus* DeBach (Hymenoptera: Aphelinidae).

The results of exposure to chlorantraniliprole + lambda-cyhalothrin, deltamethrin and lambda-cyhalothrin for *T*. *radiata* differ from those obtained by Suh et al. [33], who reported high mortality (84%) of the parasitoid *Trichogramma exiguum* Pinto & Platner (Hymenoptera: Trichogrammatidae) exposed to lambda-cyhalothrin residues; and from those obtained by Bacci et al. [34], who concluded that deltamethrin was selective for the adults of the parasitoid *Oomyzus sokolowskii* (Kurdjumov) (Hymenoptera: Eulophidae).

Among the insecticides studied here, gamma-cyhalothrin, etofenprox, azadirachtin, tebufenozide and pyriproxyfen caused lower mortality (< 25%) of the parasitoid, and were considered harmless (Class 1) to adults of *T*. *radiata* in laboratory tests. These results can be applied in the field, since the exposure conditions in the laboratory are drastic [35]; and can be recommended for IPM programs. The remaining products need to be tested under semi-field and field conditions to assess their impacts on the parasitoid.

Similar results were obtained by Brunner et al. [28] in assessing the effects of tebufenozide and azadirachtin on the parasitoid *C*. *florus* and of piriproxyfen on *A*. *melinus* adults. However, divergent results for gamma-cyhalothrin and etofenprox are reported in the literature. Haseeb et al. [36] indicated that gamma-cyhalothrin caused high mortality to adults of the parasitoid *Cotesia plutellae* (Kurdjumov) (Hymenoptera: Braconidae) when exposed to residues of this insecticide; and Moscardini et al. [37] and Giolo et al. [38] reported high mortality rates of parasitoids when exposed to residues of etofenprox.

In addition, products that were classified as harmless in the acute toxicity test allowed parasitism of the maternal and F_1_ generations, as well as emergence of parasitoids in the F_1_ and F_2_ generations. Although tebufenozide and gamma-cyhalothrin differed statistically from the control in terms of the parasitism rate of the maternal generation, they were classified as slightly harmful (Class 2), as were all other insecticides tested. The results obtained for tebufenozide diverge from those obtained by Pratissoli et al. [39], who observed no effects on the parasitism rate, viability and sex ratio of *T*. *pretiosum* in eggs of *Spodoptera frugiperda* (J.E. Smith) (Lepidoptera: Noctuidae); and by Cônsoli et al. [40] for *T*. *galloi*, who observed no effect on the parasitism capacity of females that emerged from parasitized eggs treated with tebufenozide, for the F_0_ and F_1_ generations.

The lower parasitism rate observed in females exposed to tebufenozide residue may be attributed to the mode of action of the insecticide, which belongs to the group of growth regulators. Although they are more specific for immature stages of insects, the residues may have affected the morphology and/or physiology of *T*. *radiata*, leading to lower parasitism rates compared with control. Divergent results were reported by Chao et al. [41] and Wang et al. [42], who found that tefubenozide did not affect the parasitism capacity of the parasitoid *Trichogramma japonicum* Ashmead (Hymenoptera: Trichogrammatidea) and of the parasitoid *Trichogrammatoidea bactrae* Nagaraja (Hymenoptera: Trichogrammatidea), respectively.

In agreement with data from Chen et al. [43], in this study, azadirachtin affected the parasitism capacity of the F_0_ generation, but did not affect the emergence of the F_1_ generation, and was considered slightly harmful (Class 2). Divergent results were obtained by Biondi et al. [44]; indeed, although azadirachtin was slightly harmful to adults and pupae of the Braconid wasp *Bracon nigricans* Szépligeti (Hymenoptera: Braconidae), on the same wasp this biopesticide caused significant life history trait modifications that caused delays in population growth.

The pyrethroid gamma-cyhalothrin is considered harmful (Class 4) to several natural enemies [45]. As the residue did not cause high mortality of the parasitoid tested here, this pyrethroid may have affected the morphology and/or physiology of *T*. *radiata*, decreasing the parasitism of the generation exposed to its residue, in agreement with data from Carmo et al. [45] for *Telenomus remus* (Nixon) (Hymenoptera: Scelionidae).

In this study, etofenprox was considered slightly harmful or harmless (Class 2 and 1) for generations F_0_ and F_1_, respectively. However, Souza et al. [46] obtained different results for *T*. *pretiosum*, reporting etofenprox as moderately harmful (Class 3). Goulart et al. [47] also reported that etofenprox was extremely harmful to the parasitoids *T*. *pretiosum* and *T*. *exiguum* (Class 4). These results diverge from those found in this study because Souza et al. [46] treated the host eggs before offering them to the parasitoids. This difference in methodology and in insect species used might account for the divergent the results. Goulart et al. [47] exposed the parasitoids to residues sprayed on glass dishes and then performed the parasitism test. Despite the similarity to the present study, the specimens and the substrate used to expose the maternal generation are different, which may also have led to the differences in toxicological classification.

Pyriproxyfen affected the parasitism capacity of the F_0_ and F_1_ generations, and was classified as slightly harmful (Class 2) to the F_0_ and F_1_ generations of *T*. *radiata*. Similar results were also obtained when treating *A*. *melinus* larvae with pyriproxyfen; indeed this insect growth regulator strongly impacted the juvenile survival and especially the reproductive capacity (both progeny size and sex-ratio) of the adults that developed from treated larvae [24]. Carvalho et al. [48] obtained similar results by spraying eggs of *Anagasta kuehniella* (Zeller) (Lepidoptera: Pyralidae) with pyriproxyfen and offering them to *T*. *pretiosum* to assess the effects on parasitism capacity. The authors observed a reduction in this parameter, however without a negative effect on the emergence of the F_1_ generation.

When the parasitoid pupae were sprayed, a lower emergence was observed only for dimethoate and chlorpyrifos. In addition, adults that were able to emerge from these treatments were more debilitated and died shortly after emergence, indicating that the insecticide residue may have been present on the nymphs of *D*. *citri*, since the insecticides were applied a day or two before emergence, so that the parasitoids might have come in contact with the residues as they opened the hole for emerging from the nymph. Adult parasitoids, despite being an ectoparasitoid, develop underneath the nymph [10] and emerge by making a hole in the thorax or head of the mummified nymph [49]. Dimethoate and chlorpyrifos caused low emergence of *T*. *radiata*, probably because the insecticide penetrated through the host nymphs, causing mortality of the parasitoid in the mummified body of the *D*. *citri* nymph.

The relatively low impact of pyrethroids on pupae of *T*. *radiata* observed in this study is probably attributable to protection of the pupal stage underneath the host nymph.

The insecticides that were considered harmful to the parasitoid in the acute toxicity test did not reduce adult emergence when sprayed in the pupal stage, except for the organophosphates dimethoate and chlorpyrifos, which proved to be harmful to *T*. *radiata* in the adult and pupal stages.

Other studies on selectivity of insecticides for the parasitoid *T*. *radiata* in semi-field and field conditions are necessary to investigate the impact of harmful products under these conditions. Thus, the results will be more comprehensive and useful in IPM programs for citrus, in order to combine chemical with biological control.
